# [2,2-Bis(diphenyl­phosphan­yl)propane-κ^2^ *P*,*P*′]tetra­carbonyl­chromium(0) dichloro­methane monosolvate

**DOI:** 10.1107/S1600536810043692

**Published:** 2010-10-31

**Authors:** Normen Peulecke, Stephan Peitz, Bernd H. Müller, Anke Spannenberg, Uwe Rosenthal

**Affiliations:** a Leibniz-Institut für Katalyse e. V. an der Universität Rostock, Albert-Einstein-Strasse 29a, 18059 Rostock, Germany

## Abstract

The title compound, [Cr(C_27_H_26_P_2_)(CO)_4_]·CH_2_Cl_2_, was obtained by the reaction of Ph_2_PCMe_2_PPh_2_ with Cr(CO)_6_ in refluxing toluene by substitution of two carbonyl ligands. The CrC_4_P_2_ coordination geometry at the Cr atom is distorted octa­hedral, with a P—Cr—P bite angle of 70.27 (2)°.

## Related literature

For the original synthesis of Ph_2_PCMe_2_PPh_2_, see: Hewertson & Watson (1962 ▶). For an alternative synthesis of the title compound, see: Al-Jibori & Shaw (1983 ▶). For the synthesis of Ph_2_PCMe_2_PPh_2_ and Mo or W carbonyl complexes of related ligands with different substituents at the central carbon, see: Hogarth & Kilmartin (2007 ▶). For complexation of Ph_2_PCMe_2_PPh_2 _and structural characterization of monomeric complexes of Pd or Ru, see: Barkley *et al.* (1995 ▶, 1998 ▶); Anandhi *et al.* (2003 ▶).
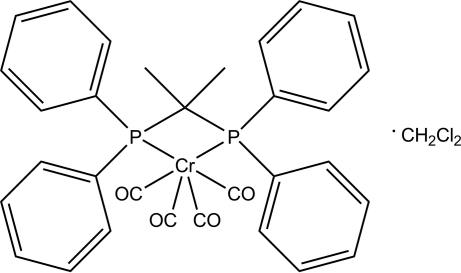


## Experimental

### 

#### Crystal data

[Cr(C_27_H_26_P_2_)(CO)_4_]·CH_2_Cl_2_
*M* *_r_* = 661.38
Triclinic, 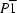

*a* = 8.9998 (5) Å
*b* = 9.4895 (5) Å
*c* = 18.3178 (9) Å
α = 99.811 (4)°
β = 94.856 (4)°
γ = 93.020 (4)°
*V* = 1532.40 (14) Å^3^
*Z* = 2
Mo *K*α radiation
μ = 0.69 mm^−1^
*T* = 150 K
0.50 × 0.50 × 0.27 mm

#### Data collection

Stoe IPDS II diffractometer
Absorption correction: numerical (*X-SHAPE* and *X-RED32*; Stoe & Cie, 2005 ▶) *T*_min_ = 0.700, *T*_max_ = 0.834
25497 measured reflections
7051 independent reflections
5824 reflections with *I* > 2σ(*I*)
*R*_int_ = 0.038

#### Refinement

*R*[*F* ^2^ > 2σ(*F* ^2^)] = 0.033
*wR*(*F* ^2^) = 0.087
*S* = 1.06
7051 reflections
372 parameters
H-atom parameters constrained
Δρ_max_ = 0.71 e Å^−3^
Δρ_min_ = −0.65 e Å^−3^

### 

Data collection: *X-AREA* (Stoe & Cie, 2005 ▶); cell refinement: *X-AREA*; data reduction: *X-AREA*; program(s) used to solve structure: *SHELXS97* (Sheldrick, 2008 ▶); program(s) used to refine structure: *SHELXL97* (Sheldrick, 2008 ▶); molecular graphics: *XP* in *SHELXTL* (Sheldrick, 2008 ▶); software used to prepare material for publication: *SHELXL97*.

## Supplementary Material

Crystal structure: contains datablocks I, global. DOI: 10.1107/S1600536810043692/cv2782sup1.cif

Structure factors: contains datablocks I. DOI: 10.1107/S1600536810043692/cv2782Isup2.hkl

Additional supplementary materials:  crystallographic information; 3D view; checkCIF report

## Figures and Tables

**Fig. 1.**
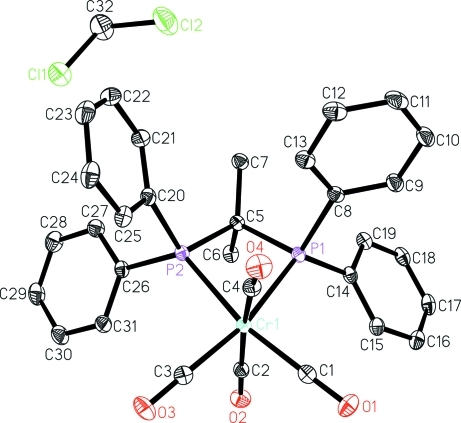
The molecular structure of the title compound showing the atom-labelling scheme. H atoms are omitted for clarity. Thermal ellipsoids are drawn at the 30% probability level.

## supplementary crystallographic information

### Comment

2,2-Bis(diphenylphosphino)propane was first prepared by cleavage of triphenylphosphine with sodium in liquid ammonia and following treatment with 2,2-dichloropropane (Hewertson & Watson, 1962). Most of the small bite-angle diphosphine complexes, of the type [*M*(CO)_4_{Ph_2_PC(R1R2)PPh_2_}] (*M* = Mo, W; R1 = H, Me, Et, Pr, allyl, R2 = Me, allyl), have been prepared *via* elaboration of the methylene backbones in [*M*(CO)_4_(Ph_2_PCH_2_PPh_2_)] (Ph_2_PCH_2_PPh_2_ = dppm) as a result of successive deprotonation and alkyl halide addition (Hogarth & Kilmartin, 2007). The above mentioned chromium complex [Cr(CO)_4_(Ph_2_PCMe_2_PPh_2_)] was prepared also by this way, but not structurally characterized yet (Al-Jibori & Shaw, 1983). Molecular structures of monomeric ruthenium (Barkley *et al.*, 1998; Anandhi *et al.*, 2003) and palladium (Barkley *et al.*, 1995) complexes of 2,2-bis(diphenylphosphino)propane are already known.

Here we describe the synthesis of the known chromium complex C_31_H_26_CrO_4_P_2_ by direct reaction of Ph_2_PCMe_2_PPh_2_ with Cr(CO)_6_. Crystals suitable for X-ray analysis were obtained from dichloromethane/methanol solution. The asymmetric unit contains one complex molecule and additionally one solvent molecule dichloromethane. The chromium center is coordinated by the chelating diphosphine Ph_2_PCMe_2_PPh_2_ and four carbonyl ligands in a distorted octahedral geometry. A bite-angle P—Cr—P of 70.27 (2)° was observed. The P—C—P angle of the complexed ligand is 92.07 (7)°. In the crystal structure, short distance of 3.807 (2) Å between the centroids of aromatic rings C14–C19 from the neighbouring molecules suggests an existence of weak π–π interactions.

### Experimental

Cr(CO)_6_ (175 mg, 0.8 mmol) was added to a solution of Ph_2_PCMe_2_PPh_2_ (309 mg, 0.75 mmol) in 20 ml of toluene and the resulting solution was stirred at reflux temperature for 72 h. Subsequently, the formed yellow solution was cooled down to 0°C and filtered. Toluene was removed in vacuum and the product was extracted with dichloromethane. The major part of dichloromethane was removed and the remaining solution was over-layered with methanol to get crystals of the title compound at -40°C, which are suitable for X-ray crystal structure analysis. The analytical data of the yellow compound correspond with those in the literature.

### Refinement

All H atoms were placed in idealized positions with d(C—H) = 0.99 (CH_2_), 0.98 (CH_3_) and 0.95 Å (CH) and refined using a riding model with *U*_iso_(H) fixed at 1.5*U*_eq_(C) for CH_3_ and 1.2*U*_eq_(C) for CH_2_ and CH.

### Crystal data

**Table:**

[Cr(C_27_H_26_P_2_)(CO)_4_]·CH_2_Cl_2_	*Z* = 2
*M**_r_* = 661.38	*F*(000) = 680
Triclinic, *P*1	*D*_x_ = 1.433 Mg m^−^^3^
Hall symbol: -P 1	Mo *K*α radiation, λ = 0.71073 Å
*a* = 8.9998 (5) Å	Cell parameters from 4481 reflections
*b* = 9.4895 (5) Å	θ = 2.2–29.6°
*c* = 18.3178 (9) Å	µ = 0.69 mm^−^^1^
α = 99.811 (4)°	*T* = 150 K
β = 94.856 (4)°	Prism, yellow
γ = 93.020 (4)°	0.50 × 0.50 × 0.27 mm
*V* = 1532.40 (14) Å^3^	

### Data collection

**Table:**

Stoe IPDS II diffractometer	7051 independent reflections
Radiation source: fine-focus sealed tube	5824 reflections with *I* > 2σ(*I*)
graphite	*R*_int_ = 0.038
ω scans	θ_max_ = 27.5°, θ_min_ = 2.2°
Absorption correction: numerical (*X-SHAPE* and *X-RED32*; Stoe & Cie, 2005)	*h* = −11→11
*T*_min_ = 0.700, *T*_max_ = 0.834	*k* = −12→12
25497 measured reflections	*l* = −23→23

### Refinement

**Table:**

Refinement on *F*^2^	Primary atom site location: structure-invariant direct methods
Least-squares matrix: full	Secondary atom site location: difference Fourier map
*R*[*F*^2^ > 2σ(*F*^2^)] = 0.033	Hydrogen site location: inferred from neighbouring sites
*wR*(*F*^2^) = 0.087	H-atom parameters constrained
*S* = 1.06	*w* = 1/[σ^2^(*F*_o_^2^) + (0.0478*P*)^2^ + 0.3172*P*] where *P* = (*F*_o_^2^ + 2*F*_c_^2^)/3
7051 reflections	(Δ/σ)_max_ = 0.001
372 parameters	Δρ_max_ = 0.71 e Å^−^^3^
0 restraints	Δρ_min_ = −0.65 e Å^−^^3^

### Special details

**Table:**

Geometry. All e.s.d.'s (except the e.s.d. in the dihedral angle between two l.s. planes) are estimated using the full covariance matrix. The cell e.s.d.'s are taken into account individually in the estimation of e.s.d.'s in distances, angles and torsion angles; correlations between e.s.d.'s in cell parameters are only used when they are defined by crystal symmetry. An approximate (isotropic) treatment of cell e.s.d.'s is used for estimating e.s.d.'s involving l.s. planes.
Refinement. Refinement of *F*^2^ against ALL reflections. The weighted *R*-factor *wR* and goodness of fit *S* are based on *F*^2^, conventional *R*-factors *R* are based on *F*, with *F* set to zero for negative *F*^2^. The threshold expression of *F*^2^ > σ(*F*^2^) is used only for calculating *R*-factors(gt) *etc*. and is not relevant to the choice of reflections for refinement. *R*-factors based on *F*^2^ are statistically about twice as large as those based on *F*, and *R*-factors based on ALL data will be even larger.

### Fractional atomic coordinates and isotropic or equivalent isotropic displacement parameters (Å^2^)

**Table:**

	*x*	*y*	*z*	*U*_iso_*/*U*_eq_	
C1	1.01536 (18)	0.20775 (19)	0.18509 (10)	0.0267 (4)	
C2	0.76174 (18)	0.13420 (18)	0.22785 (10)	0.0248 (3)	
C3	0.96144 (18)	0.26041 (19)	0.33115 (10)	0.0277 (4)	
C4	1.02766 (18)	0.46144 (19)	0.25461 (10)	0.0270 (4)	
C5	0.57483 (17)	0.44246 (17)	0.20067 (9)	0.0216 (3)	
C6	0.45608 (17)	0.31571 (19)	0.19234 (10)	0.0259 (3)	
H6A	0.3801	0.3405	0.2268	0.039*	
H6B	0.5041	0.2307	0.2038	0.039*	
H6C	0.4087	0.2955	0.1411	0.039*	
C7	0.49660 (19)	0.57332 (19)	0.18302 (10)	0.0277 (4)	
H7A	0.4490	0.5525	0.1319	0.041*	
H7B	0.5702	0.6552	0.1883	0.041*	
H7C	0.4204	0.5963	0.2176	0.041*	
C8	0.80565 (17)	0.54283 (18)	0.10865 (10)	0.0241 (3)	
C9	0.81566 (19)	0.5386 (2)	0.03282 (11)	0.0300 (4)	
H9	0.7802	0.4547	−0.0017	0.036*	
C10	0.8776 (2)	0.6569 (2)	0.00739 (12)	0.0369 (4)	
H10	0.8841	0.6533	−0.0444	0.044*	
C11	0.9293 (2)	0.7788 (2)	0.05695 (13)	0.0381 (5)	
H11	0.9712	0.8592	0.0393	0.046*	
C12	0.9204 (2)	0.7847 (2)	0.13238 (13)	0.0356 (4)	
H12	0.9558	0.8691	0.1665	0.043*	
C13	0.85938 (18)	0.66646 (19)	0.15817 (11)	0.0292 (4)	
H13	0.8544	0.6703	0.2101	0.035*	
C14	0.66313 (18)	0.26334 (18)	0.06079 (9)	0.0240 (3)	
C15	0.73759 (18)	0.14068 (19)	0.03805 (10)	0.0264 (4)	
H15	0.8206	0.1186	0.0687	0.032*	
C16	0.6919 (2)	0.0507 (2)	−0.02876 (11)	0.0316 (4)	
H16	0.7451	−0.0313	−0.0443	0.038*	
C17	0.5691 (2)	0.0798 (2)	−0.07279 (10)	0.0325 (4)	
H17	0.5380	0.0180	−0.1186	0.039*	
C18	0.4914 (2)	0.1991 (2)	−0.05008 (10)	0.0309 (4)	
H18	0.4053	0.2176	−0.0797	0.037*	
C19	0.53874 (19)	0.2914 (2)	0.01568 (10)	0.0282 (4)	
H19	0.4864	0.3744	0.0303	0.034*	
C20	0.74847 (18)	0.63022 (18)	0.34037 (9)	0.0241 (3)	
C21	0.6667 (2)	0.74999 (19)	0.33735 (11)	0.0321 (4)	
H21	0.5747	0.7410	0.3069	0.039*	
C22	0.7189 (3)	0.8825 (2)	0.37862 (12)	0.0403 (5)	
H22	0.6632	0.9640	0.3755	0.048*	
C23	0.8507 (2)	0.8967 (2)	0.42396 (12)	0.0410 (5)	
H23	0.8867	0.9880	0.4513	0.049*	
C24	0.9306 (2)	0.7781 (2)	0.42962 (11)	0.0367 (4)	
H24	1.0197	0.7869	0.4623	0.044*	
C25	0.88029 (19)	0.6458 (2)	0.38738 (10)	0.0289 (4)	
H25	0.9367	0.5648	0.3906	0.035*	
C26	0.56456 (17)	0.39289 (17)	0.35684 (9)	0.0232 (3)	
C27	0.44294 (19)	0.4714 (2)	0.37624 (11)	0.0309 (4)	
H27	0.4247	0.5550	0.3557	0.037*	
C28	0.3486 (2)	0.4280 (2)	0.42524 (12)	0.0377 (4)	
H28	0.2654	0.4816	0.4380	0.045*	
C29	0.3749 (2)	0.3072 (2)	0.45557 (11)	0.0381 (5)	
H29	0.3098	0.2776	0.4892	0.046*	
C30	0.4951 (2)	0.2296 (2)	0.43722 (11)	0.0371 (4)	
H30	0.5133	0.1469	0.4585	0.045*	
C31	0.5902 (2)	0.27148 (19)	0.38763 (10)	0.0293 (4)	
H31	0.6728	0.2170	0.3748	0.035*	
C32	0.2113 (3)	0.9291 (3)	0.33113 (16)	0.0622 (7)	
H32A	0.1155	0.8720	0.3145	0.075*	
H32B	0.1881	1.0281	0.3515	0.075*	
Cl1	0.30771 (6)	0.85575 (7)	0.40154 (4)	0.05184 (15)	
Cl2	0.31373 (9)	0.93219 (10)	0.25513 (5)	0.0796 (2)	
Cr1	0.88203 (3)	0.30677 (3)	0.242280 (15)	0.02031 (8)	
O1	1.09906 (15)	0.14688 (15)	0.14916 (8)	0.0396 (3)	
O2	0.69755 (15)	0.02391 (13)	0.21958 (8)	0.0353 (3)	
O3	1.00875 (16)	0.22861 (17)	0.38596 (8)	0.0426 (3)	
O4	1.12468 (14)	0.54764 (15)	0.26147 (9)	0.0401 (3)	
P1	0.73655 (4)	0.38536 (4)	0.14524 (2)	0.02048 (10)	
P2	0.69140 (4)	0.44708 (4)	0.29237 (2)	0.01995 (10)	

### Atomic displacement parameters (Å^2^)

**Table:**

	*U*^11^	*U*^22^	*U*^33^	*U*^12^	*U*^13^	*U*^23^
C1	0.0248 (8)	0.0285 (9)	0.0269 (9)	−0.0012 (7)	0.0000 (7)	0.0071 (7)
C2	0.0244 (7)	0.0281 (9)	0.0225 (9)	0.0037 (6)	0.0024 (6)	0.0059 (7)
C3	0.0239 (8)	0.0286 (9)	0.0303 (10)	0.0001 (6)	0.0025 (7)	0.0050 (7)
C4	0.0233 (8)	0.0305 (9)	0.0274 (9)	0.0040 (7)	0.0026 (7)	0.0052 (7)
C5	0.0195 (7)	0.0232 (8)	0.0223 (8)	−0.0007 (6)	0.0019 (6)	0.0055 (6)
C6	0.0208 (7)	0.0305 (9)	0.0255 (9)	−0.0047 (6)	0.0011 (6)	0.0045 (7)
C7	0.0262 (8)	0.0297 (9)	0.0282 (9)	0.0041 (7)	0.0018 (7)	0.0083 (7)
C8	0.0197 (7)	0.0257 (8)	0.0293 (9)	0.0016 (6)	0.0050 (6)	0.0107 (7)
C9	0.0283 (8)	0.0338 (9)	0.0312 (10)	0.0033 (7)	0.0074 (7)	0.0120 (8)
C10	0.0367 (9)	0.0418 (11)	0.0396 (11)	0.0070 (8)	0.0164 (8)	0.0207 (9)
C11	0.0294 (9)	0.0347 (10)	0.0577 (14)	0.0022 (7)	0.0145 (9)	0.0242 (10)
C12	0.0270 (8)	0.0296 (9)	0.0516 (13)	−0.0030 (7)	0.0042 (8)	0.0128 (9)
C13	0.0252 (8)	0.0287 (9)	0.0347 (10)	−0.0008 (7)	0.0035 (7)	0.0095 (8)
C14	0.0235 (7)	0.0274 (8)	0.0216 (8)	−0.0035 (6)	0.0033 (6)	0.0067 (7)
C15	0.0245 (8)	0.0299 (9)	0.0248 (9)	−0.0010 (6)	0.0042 (7)	0.0046 (7)
C16	0.0311 (9)	0.0329 (9)	0.0296 (10)	−0.0017 (7)	0.0090 (7)	0.0004 (8)
C17	0.0352 (9)	0.0396 (10)	0.0202 (9)	−0.0110 (8)	0.0033 (7)	0.0017 (8)
C18	0.0307 (9)	0.0385 (10)	0.0234 (9)	−0.0066 (7)	−0.0021 (7)	0.0101 (8)
C19	0.0276 (8)	0.0322 (9)	0.0255 (9)	−0.0007 (7)	0.0013 (7)	0.0084 (7)
C20	0.0255 (7)	0.0247 (8)	0.0226 (8)	−0.0036 (6)	0.0068 (6)	0.0049 (7)
C21	0.0390 (9)	0.0258 (9)	0.0310 (10)	0.0009 (7)	0.0028 (8)	0.0038 (8)
C22	0.0588 (12)	0.0235 (9)	0.0390 (12)	0.0011 (8)	0.0100 (10)	0.0046 (8)
C23	0.0545 (12)	0.0281 (10)	0.0363 (11)	−0.0162 (9)	0.0128 (9)	−0.0043 (8)
C24	0.0323 (9)	0.0430 (11)	0.0294 (10)	−0.0122 (8)	0.0051 (8)	−0.0055 (8)
C25	0.0268 (8)	0.0319 (9)	0.0259 (9)	−0.0030 (7)	0.0049 (7)	−0.0002 (7)
C26	0.0240 (7)	0.0244 (8)	0.0204 (8)	−0.0043 (6)	0.0026 (6)	0.0037 (7)
C27	0.0286 (8)	0.0334 (9)	0.0323 (10)	0.0017 (7)	0.0072 (7)	0.0086 (8)
C28	0.0301 (9)	0.0443 (11)	0.0390 (11)	−0.0010 (8)	0.0127 (8)	0.0047 (9)
C29	0.0386 (10)	0.0449 (11)	0.0314 (10)	−0.0105 (8)	0.0124 (8)	0.0083 (9)
C30	0.0467 (11)	0.0348 (10)	0.0326 (11)	−0.0041 (8)	0.0093 (9)	0.0136 (8)
C31	0.0338 (9)	0.0273 (9)	0.0281 (10)	−0.0001 (7)	0.0063 (7)	0.0075 (7)
C32	0.0465 (13)	0.085 (2)	0.0622 (17)	0.0223 (13)	0.0112 (12)	0.0258 (15)
Cl1	0.0416 (3)	0.0617 (4)	0.0546 (4)	0.0032 (2)	0.0064 (2)	0.0164 (3)
Cl2	0.0798 (5)	0.1007 (6)	0.0752 (5)	0.0227 (4)	0.0300 (4)	0.0472 (5)
Cr1	0.01840 (12)	0.02201 (14)	0.02079 (15)	0.00010 (9)	0.00154 (10)	0.00511 (10)
O1	0.0335 (7)	0.0421 (8)	0.0431 (8)	0.0087 (6)	0.0132 (6)	0.0005 (7)
O2	0.0371 (7)	0.0261 (7)	0.0414 (8)	−0.0056 (5)	0.0009 (6)	0.0059 (6)
O3	0.0417 (8)	0.0550 (9)	0.0330 (8)	0.0023 (6)	−0.0071 (6)	0.0186 (7)
O4	0.0281 (6)	0.0383 (7)	0.0523 (9)	−0.0088 (6)	0.0027 (6)	0.0071 (7)
P1	0.01951 (18)	0.0222 (2)	0.0203 (2)	−0.00082 (15)	0.00222 (15)	0.00564 (16)
P2	0.01915 (18)	0.0205 (2)	0.0203 (2)	−0.00140 (14)	0.00230 (15)	0.00456 (16)

### Geometric parameters (Å, °)

**Table:**

C1—O1	1.155 (2)	C16—H16	0.9500
C1—Cr1	1.8484 (19)	C17—C18	1.384 (3)
C2—O2	1.149 (2)	C17—H17	0.9500
C2—Cr1	1.8817 (17)	C18—C19	1.383 (3)
C3—O3	1.151 (2)	C18—H18	0.9500
C3—Cr1	1.8533 (19)	C19—H19	0.9500
C4—O4	1.148 (2)	C20—C25	1.391 (2)
C4—Cr1	1.8860 (17)	C20—C21	1.392 (3)
C5—C7	1.527 (2)	C20—P2	1.8347 (17)
C5—C6	1.545 (2)	C21—C22	1.388 (3)
C5—P1	1.8943 (16)	C21—H21	0.9500
C5—P2	1.8963 (17)	C22—C23	1.376 (3)
C6—H6A	0.9800	C22—H22	0.9500
C6—H6B	0.9800	C23—C24	1.381 (3)
C6—H6C	0.9800	C23—H23	0.9500
C7—H7A	0.9800	C24—C25	1.389 (3)
C7—H7B	0.9800	C24—H24	0.9500
C7—H7C	0.9800	C25—H25	0.9500
C8—C13	1.392 (3)	C26—C31	1.388 (2)
C8—C9	1.393 (3)	C26—C27	1.393 (3)
C8—P1	1.8393 (16)	C26—P2	1.8263 (16)
C9—C10	1.394 (2)	C27—C28	1.384 (2)
C9—H9	0.9500	C27—H27	0.9500
C10—C11	1.376 (3)	C28—C29	1.380 (3)
C10—H10	0.9500	C28—H28	0.9500
C11—C12	1.383 (3)	C29—C30	1.373 (3)
C11—H11	0.9500	C29—H29	0.9500
C12—C13	1.394 (2)	C30—C31	1.391 (2)
C12—H12	0.9500	C30—H30	0.9500
C13—H13	0.9500	C31—H31	0.9500
C14—C15	1.393 (3)	C32—Cl2	1.737 (3)
C14—C19	1.399 (2)	C32—Cl1	1.755 (3)
C14—P1	1.8184 (18)	C32—H32A	0.9900
C15—C16	1.385 (3)	C32—H32B	0.9900
C15—H15	0.9500	Cr1—P1	2.3644 (5)
C16—C17	1.380 (3)	Cr1—P2	2.3767 (5)
			
O1—C1—Cr1	179.44 (16)	C23—C22—C21	120.44 (19)
O2—C2—Cr1	175.10 (15)	C23—C22—H22	119.8
O3—C3—Cr1	178.39 (16)	C21—C22—H22	119.8
O4—C4—Cr1	174.55 (16)	C22—C23—C24	119.97 (18)
C7—C5—C6	108.25 (13)	C22—C23—H23	120.0
C7—C5—P1	117.15 (11)	C24—C23—H23	120.0
C6—C5—P1	109.61 (12)	C23—C24—C25	119.77 (19)
C7—C5—P2	121.62 (12)	C23—C24—H24	120.1
C6—C5—P2	106.88 (11)	C25—C24—H24	120.1
P1—C5—P2	92.07 (7)	C24—C25—C20	120.90 (18)
C5—C6—H6A	109.5	C24—C25—H25	119.6
C5—C6—H6B	109.5	C20—C25—H25	119.6
H6A—C6—H6B	109.5	C31—C26—C27	119.18 (15)
C5—C6—H6C	109.5	C31—C26—P2	119.40 (13)
H6A—C6—H6C	109.5	C27—C26—P2	121.43 (13)
H6B—C6—H6C	109.5	C28—C27—C26	120.27 (17)
C5—C7—H7A	109.5	C28—C27—H27	119.9
C5—C7—H7B	109.5	C26—C27—H27	119.9
H7A—C7—H7B	109.5	C29—C28—C27	120.18 (19)
C5—C7—H7C	109.5	C29—C28—H28	119.9
H7A—C7—H7C	109.5	C27—C28—H28	119.9
H7B—C7—H7C	109.5	C30—C29—C28	120.01 (17)
C13—C8—C9	118.82 (15)	C30—C29—H29	120.0
C13—C8—P1	119.29 (13)	C28—C29—H29	120.0
C9—C8—P1	121.74 (14)	C29—C30—C31	120.39 (18)
C8—C9—C10	120.26 (19)	C29—C30—H30	119.8
C8—C9—H9	119.9	C31—C30—H30	119.8
C10—C9—H9	119.9	C26—C31—C30	119.97 (18)
C11—C10—C9	120.31 (19)	C26—C31—H31	120.0
C11—C10—H10	119.8	C30—C31—H31	120.0
C9—C10—H10	119.8	Cl2—C32—Cl1	112.25 (14)
C10—C11—C12	120.14 (17)	Cl2—C32—H32A	109.2
C10—C11—H11	119.9	Cl1—C32—H32A	109.2
C12—C11—H11	119.9	Cl2—C32—H32B	109.2
C11—C12—C13	119.81 (19)	Cl1—C32—H32B	109.2
C11—C12—H12	120.1	H32A—C32—H32B	107.9
C13—C12—H12	120.1	C1—Cr1—C3	94.87 (8)
C8—C13—C12	120.66 (18)	C1—Cr1—C2	87.32 (7)
C8—C13—H13	119.7	C3—Cr1—C2	87.49 (7)
C12—C13—H13	119.7	C1—Cr1—C4	84.72 (7)
C15—C14—C19	118.54 (16)	C3—Cr1—C4	89.19 (7)
C15—C14—P1	118.92 (13)	C2—Cr1—C4	171.09 (7)
C19—C14—P1	122.46 (14)	C1—Cr1—P1	98.01 (6)
C16—C15—C14	120.68 (17)	C3—Cr1—P1	166.91 (6)
C16—C15—H15	119.7	C2—Cr1—P1	90.76 (5)
C14—C15—H15	119.7	C4—Cr1—P1	94.31 (5)
C17—C16—C15	120.13 (18)	C1—Cr1—P2	168.24 (6)
C17—C16—H16	119.9	C3—Cr1—P2	96.88 (6)
C15—C16—H16	119.9	C2—Cr1—P2	93.54 (5)
C16—C17—C18	119.94 (17)	C4—Cr1—P2	95.07 (5)
C16—C17—H17	120.0	P1—Cr1—P2	70.265 (16)
C18—C17—H17	120.0	C14—P1—C8	102.23 (8)
C19—C18—C17	120.19 (17)	C14—P1—C5	108.57 (7)
C19—C18—H18	119.9	C8—P1—C5	106.88 (7)
C17—C18—H18	119.9	C14—P1—Cr1	121.91 (6)
C18—C19—C14	120.47 (17)	C8—P1—Cr1	119.29 (6)
C18—C19—H19	119.8	C5—P1—Cr1	96.77 (5)
C14—C19—H19	119.8	C26—P2—C20	99.66 (7)
C25—C20—C21	118.50 (16)	C26—P2—C5	106.62 (7)
C25—C20—P2	115.71 (13)	C20—P2—C5	112.64 (7)
C21—C20—P2	125.64 (13)	C26—P2—Cr1	124.58 (6)
C22—C21—C20	120.37 (18)	C20—P2—Cr1	116.97 (5)
C22—C21—H21	119.8	C5—P2—Cr1	96.31 (5)
C20—C21—H21	119.8		

## References

[bb1] Al-Jibori, S. & Shaw, B. L. (1983). *Inorg. Chim. Acta*, **74**, 235–239.

[bb2] Anandhi, U., Holbert, T., Lueng, D. & Sharp, P. R. (2003). *Inorg. Chem.* **42**, 1282–1295. 10.1021/ic025987f 12588167

[bb3] Barkley, J., Ellis, M., Higgins, S. J. & McCart, M. K. (1998). *Organometallics*, **17**, 1725–1731.

[bb4] Barkley, J. V., Grimshaw, J. C., Higgins, S. J., Hoare, P. B., McCart, M. K. & Smith, A. K. (1995). *J. Chem. Soc. Dalton Trans.* pp. 2901–2908.

[bb5] Hewertson, W. & Watson, H. R. (1962). *J. Chem. Soc.* 1490–1494.

[bb6] Hogarth, G. & Kilmartin, J. (2007). *J. Organomet. Chem.* **692**, 5655–5670.

[bb7] Sheldrick, G. M. (2008). *Acta Cryst.* A**64**, 112–122. 10.1107/S0108767307043930 18156677

[bb8] Stoe & Cie (2005). *X-SHAPE*, *X-RED32* and *X-AREA* Stoe & Cie, Darmstadt, Germany.

